# An *In Vitro* Chicken Gut Model Demonstrates Transfer of a Multidrug Resistance Plasmid from *Salmonella* to Commensal *Escherichia coli*

**DOI:** 10.1128/mBio.00777-17

**Published:** 2017-07-18

**Authors:** Roderick M. Card, Shaun A. Cawthraw, Javier Nunez-Garcia, Richard J. Ellis, Gemma Kay, Mark J. Pallen, Martin J. Woodward, Muna F. Anjum

**Affiliations:** a Department of Bacteriology, Animal and Plant Health Agency, New Haw, Addlestone, Surrey, United Kingdom; b Central Sequencing Unit, Animal and Plant Health Agency, New Haw, Addlestone, Surrey, United Kingdom; c Microbiology and Infection Unit, Warwick Medical School, University of Warwick, Coventry, United Kingdom; d Food and Nutritional Sciences Department, University of Reading, Whiteknights, Reading, United Kingdom; CEH-Oxford

**Keywords:** *Escherichia coli*, *Salmonella*, antimicrobial resistance, enteric pathogens, horizontal gene transfer, plasmids

## Abstract

The chicken gastrointestinal tract is richly populated by commensal bacteria that fulfill various beneficial roles for the host, including helping to resist colonization by pathogens. It can also facilitate the conjugative transfer of multidrug resistance (MDR) plasmids between commensal and pathogenic bacteria which is a significant public and animal health concern as it may affect our ability to treat bacterial infections. We used an *in vitro* chemostat system to approximate the chicken cecal microbiota, simulate colonization by an MDR *Salmonella* pathogen, and examine the dynamics of transfer of its MDR plasmid harboring several genes, including the extended-spectrum beta-lactamase *bla*_CTX-M1_. We also evaluated the impact of cefotaxime administration on plasmid transfer and microbial diversity. Bacterial community profiles obtained by culture-independent methods showed that *Salmonella* inoculation resulted in no significant changes to bacterial community alpha diversity and beta diversity, whereas administration of cefotaxime caused significant alterations to both measures of diversity, which largely recovered. MDR plasmid transfer from *Salmonella* to commensal *Escherichia coli* was demonstrated by PCR and whole-genome sequencing of isolates purified from agar plates containing cefotaxime. Transfer occurred to seven *E. coli* sequence types at high rates, even in the absence of cefotaxime, with resistant strains isolated within 3 days. Our chemostat system provides a good representation of bacterial interactions, including antibiotic resistance transfer *in vivo*. It can be used as an ethical and relatively inexpensive approach to model dissemination of antibiotic resistance within the gut of any animal or human and refine interventions that mitigate its spread before employing *in vivo* studies.

## IMPORTANCE

The spread of antimicrobial resistance presents a grave threat to public health and animal health and is affecting our ability to respond to bacterial infections. Transfer of antimicrobial resistance via plasmid exchange is of particular concern as it enables unrelated bacteria to acquire resistance. The gastrointestinal tract is replete with bacteria and provides an environment for plasmid transfer between commensals and pathogens. Here we use the chicken gut microbiota as an exemplar to model the effects of bacterial infection, antibiotic administration, and plasmid transfer. We show that transfer of a multidrug-resistant plasmid from the zoonotic pathogen *Salmonella* to commensal *Escherichia coli* occurs at a high rate, even in the absence of antibiotic administration. Our work demonstrates that the *in vitro* gut model provides a powerful screening tool that can be used to assess and refine interventions that mitigate the spread of antibiotic resistance in the gut before undertaking animal studies.

## INTRODUCTION

Chickens (*Gallus gallus*) are a source of human infection by zoonotic pathogens such as *Salmonella enterica*, *Campylobacter* spp., and *Escherichia coli* (1, 2). The chicken cecum appears to be a rich source of bacteria, including pathogens (3). The gut microbiota helps protect chickens from colonization by pathogens (4), but this can be weakened through administration of antibiotics that perturb the gut bacterial community, resulting in steep declines in the abundance and diversity of gut bacteria (5–11). Antibiotic administration can also select for antimicrobial resistance (AMR) genes, which may be carried by commensals or pathogens (4, 5, 12). The transfer of AMR genes between bacteria and the spread of resistance have implications for human and animal health (13, 14). Dissemination of resistance via plasmids harboring multiple AMR genes, including extended-spectrum beta-lactamases (ESBLs), is of particular therapeutic relevance and has been demonstrated between different bacterial genera on a single farm (15) and between animal and human strains of *E. coli* (16, 17).

The potential for dissemination of AMR via plasmid conjugation in the chicken cecal microbiota has not been fully defined. Various *in vitro* chemostat models have been developed to investigate the gut microbiota of humans and animals (18, 19). These models seek to simulate the physiological conditions encountered in the gut (e.g., pH and temperature) and employ culture media that support diverse bacterial communities, resembling those found *in vivo*. The models vary in complexity, with some consisting of a single batch fermentation vessel run for 24 to 48 h, whereas others use one or more vessels in series and employ a continuous flow system to introduce fresh media, allowing the system to be run for days or weeks; most monitor changes in a handful of bacteria using mainly culture-based methods (18, 19). Chemostats provide useful screening tools to examine the effects of interventions on the microbiota under controlled experimental conditions without the ethical restrictions associated with human and animal trials. Such models have been used to investigate the impact of antibiotics on proliferation of *Clostridium difficile* in a human gut model (20), the transfer of AMR gene-harboring plasmids from avian *E. coli* to a limited number of human *E. coli* clones in a human gut model (17), the production of metabolites such as short-chain fatty acids in human, chicken, and pig microbiota (21, 22), and the impact of dietary elements on human microbiota (23, 24).

In this study, we report the development of an *in vitro* chemostat system that aims to approximate the chicken cecal microbiota and use it to demonstrate the effect of infection of the chicken with a multidrug-resistant (MDR) *Salmonella* and ensuing antibiotic administration. *Salmonella* colonization was simulated by inoculation of a strain of *Salmonella enterica* serovar Typhimurium, carrying the MDR IncI1 plasmid pIFM3844 that harbors three AMR genes, including the ESBL gene *bla*_CTX-M1_, and that readily transfers between bacteria of the same and related species on farms (15). Samples were withdrawn at regular intervals over the time period that the chemostat vessels were run and analyzed for changes in total bacterial diversity using culture-independent methods, as well as for enumeration of selected bacteria by culture. An important aspect of this study was to determine whether the presence of a zoonotic pathogen in the chicken ceca harboring an MDR plasmid results in transfer and proliferation of this plasmid in commensal bacteria that may result in a significant increase in the reservoir of resistant bacteria and their possible transfer through the food chain to affect humans. The results have a wider implication in that they demonstrate how MDR plasmids may proliferate and disseminate in the gut environment of any animal, including humans.

## RESULTS

### Establishing the *in vitro* gut model and cecal microbiota profiling.

The gut model was seeded with cecal samples collected from *Salmonella*-free chickens reared under experimental conditions in a biosecure environment. To assess the total bacterial diversity using culture-independent methods, microbial profiling by amplification and pyrosequencing of the 16S rRNA gene was performed on the seeded cecal samples, and examined using QIIME (25). This showed that the phylum *Firmicutes* dominated in these cecal samples (average relative abundance of 95.17%), comprised mainly of members of the family *Ruminococcaceae* (average relative abundance of 56.81%). Other bacterial phyla were present at low abundance, as were reads unassigned to any bacteria (~0.42%; see Tables S1 and S2 in the supplemental material).

10.1128/mBio.00777-17.3 TABLE S1  Relative abundance of sequences taxonomically classified to phyla. Download TABLE S1, XLSX file, 0.01 MB. Copyright © 2017 Card et al. 2017 Card et al. This content is distributed under the terms of the Creative Commons Attribution 4.0 International license.

10.1128/mBio.00777-17.4 TABLE S2  Relative abundance of sequences taxonomically classified to family or the next highest possible resolution level (order) in cecal samples. Download TABLE S2, XLSX file, 0.01 MB. Copyright © 2017 Card et al. 2017 Card et al. This content is distributed under the terms of the Creative Commons Attribution 4.0 International license.

**FIG 1:**
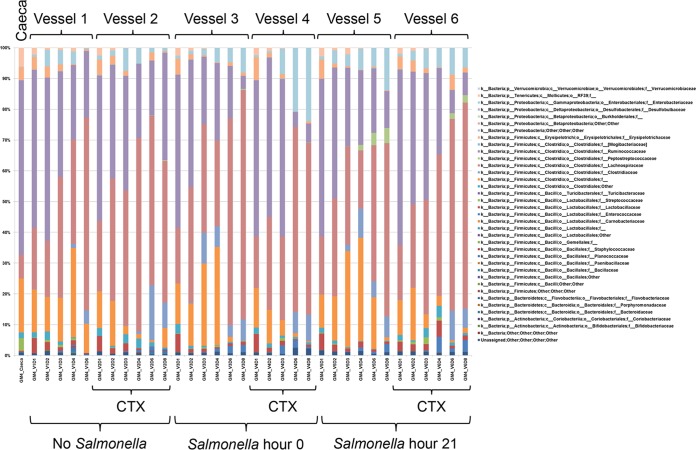
Microbial profiles obtained in experiment 4 showing responses to cefotaxime (CTX) administration and time point. Populations are presented as relative abundance of sequences taxonomically classified to family or the next highest possible resolution level (order, class, or phylum), and the isolates are identified by the vessel (V) number and day of isolation (D). The data presented in this figure are also given in Table S3 in the supplemental material.

Microbial profiles of the total bacterial population were also generated from samples withdrawn at the different time points for each vessel in the four gut model experiments performed after seeding with cecal contents. The culture-independent methods examined these bacterial communities, and the microbial profiles obtained from the four experiments were summarized as the relative abundance of sequences taxonomically classified to family or next highest possible resolution level (order, class, or phylum) (Table S3). Similar results were obtained in the four experiments (see below), with microbial profiles obtained in experiment 4 shown in Fig. 1 as an exemplar of the results obtained. In all vessels, a diverse bacterial population was present, which altered over time and in response to cefotaxime administration (Fig. 1). Importantly, no single taxon was seen to dominate. To identify statistically significant changes in the bacterial communities, the 16S rRNA metagenomic data from all four experiments were analyzed using QIIME and structured to allow comparison by experiment number, time point, cefotaxime administration, and *Salmonella* inoculation. The results showed a significant but small difference in microbial community composition between experiments (*P* = 0.002 and *R*^2^ = 0.094 by Adonis analysis). These differences arose from dissimilar abundances in the input cecal material. Indeed, of the 18 operational taxonomic units (OTUs) that were significantly different in experiments, 8 were not detected in one or more cecal preparations. Importantly, there were no significant differences between experiments in the alpha diversity indices estimating bacterial species richness (Chao1, *P* = 0.789) or estimating bacterial community evenness (Shannon, *P* = 0.425) (Table 1 and Fig. S1), indicating reproducibility and consistency in the capability of the *in vitro* system to maintain diverse bacterial populations.

10.1128/mBio.00777-17.5 TABLE S3  Relative abundance of sequences taxonomically classified to family or the next highest possible resolution level (order, class, or phylum) for experiments 1 (GM1), 2 (GM2), 3 (GM3), and 4 (GM4). The vessels (V) and isolation time (in days [D]) are identified. *Salmonella* inoculation and cefotaxime administration are indicated. Download TABLE S3, XLSX file, 0.03 MB. Copyright © 2017 Card et al. 2017 Card et al. This content is distributed under the terms of the Creative Commons Attribution 4.0 International license.

10.1128/mBio.00777-17.1 FIG S1  (a) Chao1 alpha diversity plots for experiment number, time point, *Salmonella* inoculation, and cefotaxime administration. The significance of differences in Chao1 indices by each categorical variable is given. (b) Shannon alpha diversity plots for experiment number, time point, *Salmonella* inoculation, and cefotaxime administration. The significance of differences in Shannon indices by each categorical variable is given. Download FIG S1, PDF file, 0.1 MB. Copyright © 2017 Card et al. 2017 Card et al. This content is distributed under the terms of the Creative Commons Attribution 4.0 International license.

**TABLE 1:** Significance in differences between alpha and beta diversity indices for the variables (experiment, time point, *Salmonella* inoculation, and cefotaxime administration)

Variable examined	Alpha diversity			Beta diversity (weighted UniFrac) by Adonis analysis	
	Statistical test	*P* value for alpha diversity index:		*P* value	*R*^2^ value
		Chao1	Shannon		
Experiment	Kruskal-Wallis	0.789	0.425	0.002	0.094
Time point	Kruskal-Wallis	<0.001	<0.001	0.001	0.359
*Salmonella* inoculation	Kruskal-Wallis	0.956	0.392	0.374	0.026
Cefotaxime administration	Mann-Whitney (two-tailed)	0.019	<0.001	0.001	0.134

**FIG 2:**
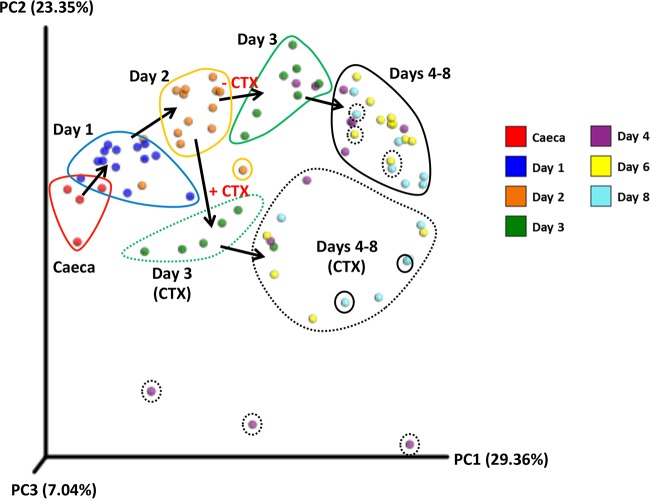
Three-dimensional principal-coordinate analysis plot of weighted UniFrac beta diversity. Samples are colored by time point as indicated in the color key. Samples at the same time point were circled manually, and the black arrows indicate the progression of time. Cefotaxime addition (+ CTX; dashed encircling) or absence of cefotaxime (− CTX; solid encircling) is shown. PC1, principal coordinate 1.

There was, however, a significant change in the bacterial community over the course of the experiment (*P* = 0.001 and *R*^2^ = 0.359 by Adonis analysis) and significant differences in the Chao1 (*P* < 0.001) and Shannon (*P* < 0.001) alpha diversity indices (Table 1 and Fig. S1). At days 1 and 2, there was an increase in alpha diversity indices above that of the input ceca, but after day 4, both indices decreased below initial levels, and these indices stabilized from day 6 onwards, with little change in the alpha and beta diversity in the bacterial community thereafter (Fig. 2 and Fig. S1). These results indicated that there was a temporary increase, followed by a decrease in community richness and abundance, followed by stability in these parameters. Changes in the bacterial communities during the course of the experiment were further investigated by three-dimensional principal-coordinate analysis of the weighted UniFrac beta diversity (Fig. 2). The majority of OTUs with a significant decrease in abundance were assigned to taxa (class or family) of obligate anaerobes such as *Clostridiales*, *Lachnospiraceae*, and *Ruminococcaceae*, although certain OTUs of *Lachnospiraceae* increased significantly. Other OTUs with a significant increase in abundance were mainly from families of facultative or aerotolerant anaerobes, such as *Clostridiaceae*, *Enterococcaceae*, and *Enterobacteriaceae*. The two OTUs from *Enterobacteriaceae* with a significant increase in abundance were not assigned to a genus by the QIIME pipeline; furthermore, there was no significant change in OTUs from the genus *Escherichia*.

### Modeling the impact of *Salmonella* inoculation on the cecal microbiota.

**FIG 3:**
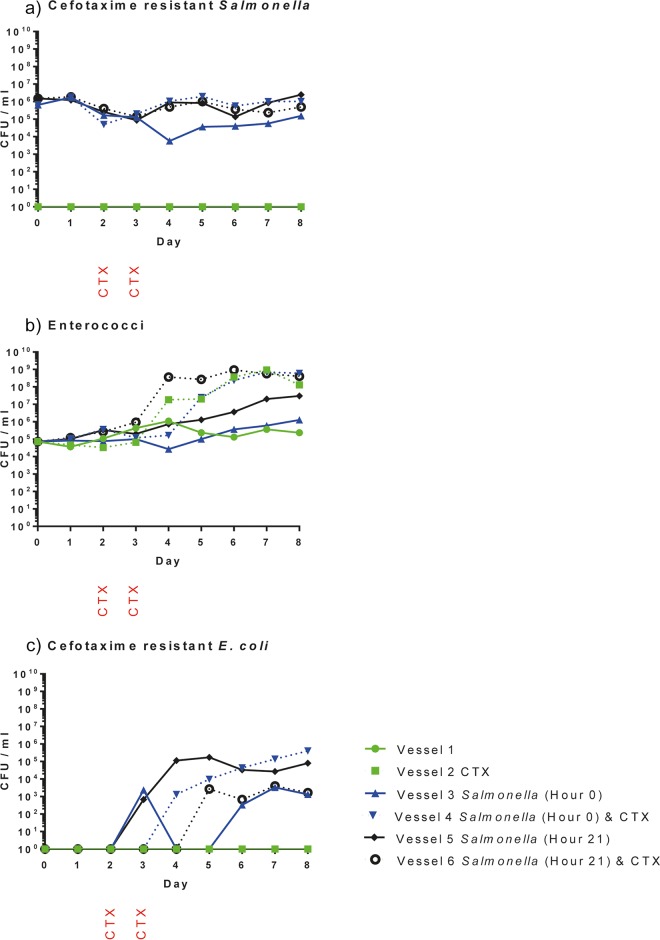
Quantitative bacteriology results for experiment 4 showing the number of CFU per milliliter of culture for presumptive cefotaxime-resistant *Salmonella* (Rambach agar) (a), enterococci (UTI Brilliance agar) (b), and cefotaxime-resistant *E. coli* (Rambach agar) (c). Counts obtained from each of the six vessels employed in the experiment are shown as presented in the symbol key. *Salmonella* was inoculated into vessels 3 and 4 at hour 0 and into vessels 5 and 6 at hour 21. Cefotaxime (CTX) was administered on the second and third day to vessels 2, 4, and 6 as indicated.

In experiments 3 and 4, 10^7^ CFU of *Salmonella* strain B3844 were inoculated into four gut model vessels per experiment (two vessels at hour 0 and two vessels at hour 21). Enumeration of the *Salmonella* by culture showed that it was maintained in all inoculated vessels at ~10^4^ to 10^6^ CFU/ml for 8 days. Similar results were obtained in both experiments, and results for experiment 4 are shown in Fig. 3a. The cecal samples used to inoculate vessels contained no *Salmonella* detected by culture on Rambach agar.

Further analysis of the microbial profile data showed no significant difference in the diversities of the bacterial communities between *Salmonella*-free chickens and those inoculated with *Salmonella* at either hour 0 or hour 21, measured by UniFrac beta diversity (*P* = 0.374 and *R*^2^ = 0.026 by Adonis analysis) (Table 1), nor any significant difference in other diversity indices such as the Chao1 (*P* = 0.956) or Shannon (*P* = 0.392) alpha diversity (Fig. S1). However, one OTU (family *Peptostreptococcaceae*) was identified with a significantly increased abundance following *Salmonella* inoculation. Three-dimensional principal-coordinate analysis of the weighted UniFrac beta diversity revealed no clustering of samples by *Salmonella* administration (Fig. S2).

10.1128/mBio.00777-17.2 FIG S2  Three-dimensional principal-coordinate analysis plot of weighted UniFrac beta diversity colored by *Salmonella* inoculation group, as indicated in the key. Samples have been grouped by day and cefotaxime administration as described in the legend to Fig. 2. Download FIG S2, TIF file, 0.1 MB. Copyright © 2017 Card et al. 2017 Card et al. This content is distributed under the terms of the Creative Commons Attribution 4.0 International license.

### Modeling the impact of cefotaxime administration on the cecal microbiota.

Microbial profiling and QIIME analysis showed that bacterial communities in cefotaxime-treated vessels showed significant decreases in species richness (Chao1 alpha diversity; *P* = 0.019) and community evenness (Shannon alpha diversity; *P* < 0.001) compared to non-antibiotic-treated vessels (Table 1 and Fig. S1). Furthermore, bacterial diversity measured by Adonis analysis comparing cefotaxime (CTX)-treated vessels with nontreated vessels using the weighted UniFrac beta diversity indicated that there were significant changes to the bacterial community after cefotaxime was administered (*P* = 0.001 and *R*^2^ = 0.134; Table 1). This change in the bacterial community was distinctly different to that observed in vessels not administered CTX, as illustrated in the principal-coordinate analysis plots (Fig. 2). Furthermore, following cefotaxime administration, 10 OTUs were significantly more abundant, including 2 *Enterococcus* OTUs, while 17 OTUs decreased in abundance (Table S4). Alterations in enterococcal populations were also seen by culture, confirming the microbial profiling data. In experiment 4, prior to cefotaxime administration, there were approximately 10^4^ to 10^5^ CFU/ml enterococci on cefotaxime-containing plates, but after cefotaxime treatment, this increased to ~10^8^ to 10^9^ CFU/ml (Fig. 3b). Increases in enterococci after cefotaxime administration were also seen in experiments 2 and 3 (not shown). The same effect was not observed in the microbial profiling or bacterial enumeration data from the three vessels not treated with cefotaxime (Fig. 3b). As expected, cefotaxime administration had no effect on *Salmonella* numbers (Fig. 3a).

10.1128/mBio.00777-17.6 TABLE S4  OTUs with significant changes in abundance following cefotaxime administration. Download TABLE S4, XLSX file, 0.01 MB. Copyright © 2017 Card et al. 2017 Card et al. This content is distributed under the terms of the Creative Commons Attribution 4.0 International license.

### Transfer of a multidrug-resistant plasmid from *Salmonella* to commensal *E. coli*.

In both experiments 3 and 4, culture on cefotaxime selective plates did not detect any presumptive *E. coli* resistant to cefotaxime in the cecal contents or at any time point in the two vessels to which *Salmonella* was not added. In experiment 4, on the third day, cefotaxime-resistant presumptive *E. coli* bacteria were detected on the Rambach agar plates supplemented with cefotaxime from two of the four vessels into which *Salmonella* had been inoculated (Fig. 3c). By day 6, cefotaxime-resistant *E. coli* bacteria were present in all vessels inoculated with *Salmonella* and remained present until the final day of the experiment (day 8). Similarly, in experiment 3, cefotaxime-resistant *E. coli* bacteria were observed in all vessels containing *Salmonella* by day 8 (not shown). Plasmid transfer rates for experiment 4 were calculated by using the endpoint bacterial enumeration method (26) with day 8 counts and ranged from 2.2 × 10^−9^ to 6.4 × 10^−10^.

All presumptive *Salmonella* isolates (*n* = 13) recovered from Rambach agar plates were verified as *Salmonella* by matrix-assisted laser desorption ionization–time of flight mass spectrometry (MALDI-TOF MS) and were PCR positive for the pIFM3844 plasmid and the *bla*_CTX-M1_ gene it harbors (Table S5). The presumptive enterococcal isolates (*n* = 6) recovered on day 5 of experiment 4 were verified as *Enterococcus faecium* by MALDI-TOF MS, and all of these isolates were PCR negative for *bla*_CTX-M1_ and pIFM3844 (Table S5). The presumptive *E. coli* isolates recovered from cefotaxime-containing plates in experiments 3 (*n* = 11 isolates) and 4 (*n* = 23 isolates) were verified as *E. coli* by MALDI-TOF MS (Table S5) and were PCR positive for *bla*_CTX-M1_ and pIFM3844 (Table S5). Presumptive *E. coli* bacteria recovered from plates without cefotaxime in experiments 3 (*n* = 6 isolates) and 4 (*n* = 12 isolates) were PCR negative for *bla*_CTX-M1_ and pIFM3844 (Table S5).

10.1128/mBio.00777-17.7 TABLE S5  Summary of isolates recovered from quantitative bacteriology plates, including experiment number, MALDI-TOF MS identification, and PCR results for plasmid pIFM3844 and *bla*_CTX-M1_. The culture media and supplements used were Rambach (RAM), Brilliance UTI (UTI), and cefotaxime (CTX). Download TABLE S5, XLSX file, 0.01 MB. Copyright © 2017 Card et al. 2017 Card et al. This content is distributed under the terms of the Creative Commons Attribution 4.0 International license.

10.1128/mBio.00777-17.8 TABLE S6  Sequence types of the nine commensal *E. coli* clones sequenced in this study. Download TABLE S6, XLSX file, 0.01 MB. Copyright © 2017 Card et al. 2017 Card et al. This content is distributed under the terms of the Creative Commons Attribution 4.0 International license.

### Whole-genome sequencing of commensal *E. coli* transconjugants.

**FIG 4:**
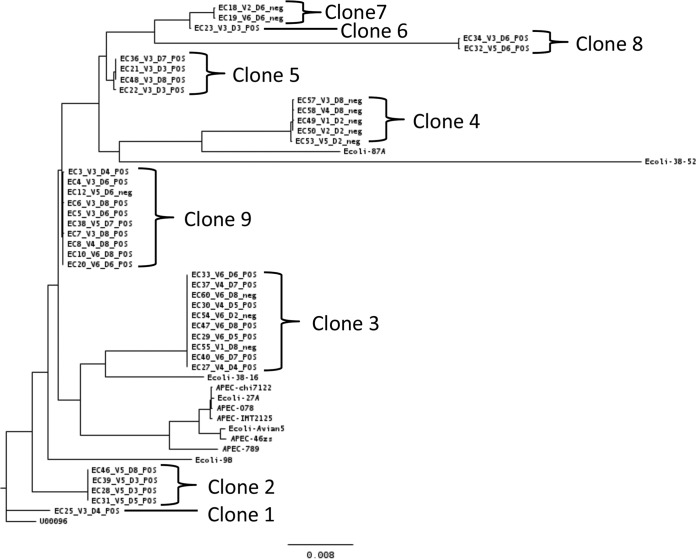
Maximum likelihood phylogenetic tree generated from core genome single nucleotide polymorphisms in RAxML. The tree contains the 39 sequenced commensal *E. coli* isolates recovered from gut model experiments 3 (clones 7 and 9) and 4 (clones 1, 2, 3, 4, 5, 6, and 8 and isolate EC38 in clone 9). *Salmonella* was inoculated into vessel 3 (V3) and V4 at hour 0 and into vessels V5 and V6 at hour 21. Cefotaxime (CTX) was administered on the second and third day to vessels V2, V4, and V6. Also included in the tree are 12 reference *E. coli* isolates from poultry comprising commensal, ESBL-harboring, or avian pathogenic *E*. *coli* (APEC) isolates. The bar indicates 0.008 nucleotide substitutions per position.

To compare the commensal *E. coli* present in the chicken cecal content to transconjugants recovered by culture and to determine whether pIFM3844 had transferred into one or more different *E. coli* clones, we performed whole-genome sequencing on 39 isolates from experiments 3 and 4. Isolates were recovered from plates with cefotaxime (*n* = 28) or without cefotaxime (*n* = 11). The maximum likelihood tree based on core genome single nucleotide polymorphisms (SNPs) showed nine subclades of *E. coli*, which we have called “clones” due to the high sequence similarity in the core genome between members of a clone, and these clones represent different *E. coli* present in the chicken microbiota in the two experiments (Fig. 4). Each clone was of a different sequence type (ST); STs were assigned to isolates from five clones, two clones had STs that were not represented in the database, and STs for two clones were assigned only provisionally due to poor sequence quality in one or more multilocus sequence type (MLST) genes (Table S6). In the phylogenetic tree constructed (Fig. 4), these 39 isolates did not cluster with the 12 published genomes of *E. coli* recovered from poultry.

The pIFM3844 plasmid was present in some isolates from clones 3 and 9, but absent in all isolates from clones 4 and 7 (Fig. 4). For all other clones, pIFM3844 was present in every isolate sequenced (Fig. 4). Isolates from clone 9 were recovered from experiments 3 and 4, whereas all other clones were recovered from one experiment only. As summarized in Fig. 4, isolates of the same clone were recovered on different days from the same vessel (clones 2, 3, 5, and 9) and/or recovered from different vessels of the same experiment (clones 3, 4, 7, 8, and 9).

The AMR gene content of each sequenced isolate was examined by SeqFinder (27). The ESBL gene *bla*_CTX-M1_ was identified in all 28 sequenced isolates that were PCR positive for pIFM3844 and *bla*_CTX-M1_, as expected. Although most commensal *E. coli* isolates did not harbor any other AMR genes, one isolate carried 11 AMR genes and three others harbored one additional AMR gene (not shown).

## DISCUSSION

We have developed an *in vitro* chemostat system that aims to simulate the chicken cecal microbiota and can be used to predict the effect of infection in chickens with multidrug-resistant *Salmonella* and ensuing antibiotic administration. To accomplish this, cecal contents from chickens hatched and reared in biosecure accommodation were used to ensure the initial absence of *Salmonella*. Microbial profiling of the uncultured cecal microbiota from these chickens showed it to be dominated by *Firmicutes*, whereas members of the phylum *Bacteroidetes* were absent. N. O. Kaakoush et al. (28) have classified the chicken microbiota into enterotypes 1 to 4; the microbiota of our biosecure chickens resembled enterotype 1, as it was dominated by *Firmicutes*. However, diversity at the family and genus level in our birds was different from that described for enterotype 1. Unlike many other farm animals, chickens are hatched and raised without contact with their parents and thus acquire the majority of their microbiota from the environment, although vertical transmission from the parent to the offspring (*in ovo* or from shell contamination) can occur for certain bacteria, including *Salmonella*. Thus, the biosecure accommodation would be expected to provide a considerably cleaner environment than that present on, for example, commercial broiler chicken farms, thereby limiting the diversity of bacteria available for colonization of the gut in birds used in our experiments.

Although the *in vitro* model is a chemostat system that can only be a surrogate of the chicken gut, it nevertheless enabled culture of a diverse bacterial community, representative of the gut, for 8 days in a reproducible manner. The bacterial community did change over this period, as assessed by microbial profiling of the uncultured microbiota, which showed that there was an initial reduction in the overall diversity and in some OTUs for obligate anaerobes; OTUs for several facultative or aerotolerant anaerobes increased significantly with time. However, importantly, the community appeared to be stabilizing by day 6 and did not come to be dominated by a single taxon. The changes in bacterial community we observed reflect the differing abilities of bacteria to grow under the culture conditions used, and such changes in bacterial community during a chemostat experiment are widely reported and not unexpected (17, 21–24, 29). Indeed, analysis using culture-independent methods, as performed in our study, is more likely to reveal these changes, since it provides much greater insight into microbial communities present in chemostat models than that offered by many other methods that have been used to examine chemostat bacterial populations, such as short-chain fatty acid production, detection of selected bacterial taxa by culture or PCR, fluorescence *in situ* hybridization, and qualitative methods such as denaturing gradient gel electrophoresis (18). We believe that the methods mentioned above do not provide a sufficiently accurate and detailed picture of the microbiota, which will emerge as more studies of *in vitro* gut models use culture-independent methods to understand the true diversity of microbial communities present in their models. Nevertheless, despite some changes seen in their microbial communities, these models will remain an invaluable and ethical tool which can be used as an effective surrogate to systematically investigate “real-life” scenarios, such as consequences of infection of the chicken with MDR *Salmonella* using controlled experimental models, and for the ensuing study of interventions that may affect the chicken microbiota. Importantly, the scenario modeled in our experiment has wider implications, as it provides insight not only into dissemination of an MDR plasmid from a pathogen to a commensal within the chicken microbiota, but possibly in any gut environment.

A limitation of all gut models is the recognition that many bacterial species have fastidious growth requirements that can be difficult to replicate *in vitro* (3, 4, 18), hence some differences between the inoculating ceca and the bacteria within the chemostat vessels is expected. Also, there is considerable variability and diversity in nature, as has been reported for the *in vivo* chicken microbiota (3, 4, 28, 30), even from chickens reared under strictly controlled conditions (31), so some variability is expected between batches of inoculating ceca. Finally, culture-independent metagenomic studies have repeatedly demonstrated that microbial diversity is highly variable over time, between populations, and due to influences from the external environment (e.g., diet), defying the concept of a stable core (32).

In this study, the model was used to examine how the chicken cecal microbiota responds to invasion by the zoonotic pathogen *Salmonella enterica* serovar Typhimurium and the impact of antibiotic administration. The results closely parallel those seen in studies performed *in vivo*, which further validate the use of this model to study such scenarios. For example, administration of cefotaxime to the model resulted in significant decreases in alpha diversity, an effect commonly reported for antibiotic administration and observed *in vivo* in chickens administered penicillin (9), tetracycline (7), or streptomycin (7), in rats administered cefotaxime (10), and in humans administered antibiotics, including beta-lactams (11). Also, inoculation of *Salmonella* into the model had no significant impact on bacterial diversity or community composition, except an increase in one OTU assigned to the family *Peptostreptococcaceae*. Importantly, these results parallel the results of studies of newly hatched chicks (33, 34) and 16-week-old laying hens (35) which have shown that infection with *Salmonella enterica* serovar Enteritidis induces minor but not significant alterations to the composition of the microbiota. One study with young chicks (36), however, reported that infection with S. Enteritidis significantly reduced microbial diversity. Interestingly, that study noted a concomitant significant increase in the abundance of several bacterial groups, including the family *Peptostreptococcaceae* (36).

We observed an increase in enterococci following cefotaxime administration by both culture (Fig. 3b) and microbial profile (see Table S4 in the supplemental material). Similarly, M. V. Tulstrup et al. (10) reported a significant increase in *Enterococcaceae* in the ilea and ceca of rats following cefotaxime treatment. Enterococci have intrinsic resistance to cephalosporins (37) and would be unaffected by the concentration of cefotaxime administered (20 mg/liter) in this study. Therefore, the observed increase is likely to be a consequence of selection of enterococci over other members of the flora, which were sensitive to cefotaxime. Enterococci remained at high abundance after antibiotic administration had ceased, suggesting a potential for a longer term alteration in the microbiota as a consequence of antibiotic administration. The importance of such “collateral effects” of antibiotics on the wider microbiota are becoming increasingly recognized (6). For example, we have reported that ciprofloxacin administration in humans leads to an increase in ciprofloxacin-resistant *Veillonella* in the saliva that is sustained for 12 months (5).

The spread of antibiotic resistance determinants by plasmids presents a significant risk to public and animal health (13, 14). In this study, we have used the gut model to demonstrate the transfer of a plasmid harboring multidrug resistance from a *Salmonella* isolate to commensal *E. coli* naturally resident in healthy chicken ceca; this model may possibly replicate the on-farm scenario (15) and those encountered in other animals or even the human gut. The plasmid transfer rate of 10^−9^ to 10^−10^ was high relative to the established values of 10^−9^ to 10^−18^ (26, 38) and occurred in all four vessels inoculated with *Salmonella* in two replicate experiments, irrespective of cefotaxime administration. By employing the chemostat system for 8 days, rather than shorter periods used for some *in vitro* models (reviewed in references 18 and 19), we were able to demonstrate a consistent and reproducible plasmid transfer response within this time frame. Using an *in vitro* human cecum model, A. Smet et al. (17) demonstrated the transfer of a plasmid carrying an ESBL gene from avian to human *E. coli* in both the presence and absence of cefotaxime, at transfer rates with the same order of magnitude as we report here.

Plasmid transfer rates are classically measured *in vitro* using pure cultures of donor and recipient at high cell densities (27). However, pure culture conditions do not accurately reproduce the bacterial communities in the gut of an animal host. Indeed, transfer rates can be considerably higher in heterogeneous bacterial communities (39). Another advantage of our model is that it comprised a diverse mixture of bacteria that sufficiently recreates the gut microbiota encountered *in vivo* so that neither bacterial donors nor recipients are dominant members. However, the model does not provide an exact facsimile of the cecum, and plasmid transfer can occur at a higher rate in well-mixed liquid cultures, such as that used in the model, than in animal intestines (17, 38), although others have reported higher rates in the gut than *in vitro* (40, 41). Another consideration when using this model is the absence of any contribution from the host, such as provision of cellular attachment sites, inflammatory responses, which can contribute to an increase of plasmid transfer (42), or secretion of factors that can reduce bacterial conjugation, as has been observed with Caco-2 cells (43). The development of *in vitro* gut models that enable the coculture of human and microbial cells shows promise, but to date, only cultures containing one or two bacterial species have been assessed (44, 45), and their capacity to maintain the complex microbiota of the gut remains unreported.

The multidrug resistance plasmid pIFM3844 transferred to seven of the nine *E. coli* clones identified in the chicken microbiota, considerably more than the two strains reported by A. Smet et al. (17). *E. coli* transconjugants harboring pIFM3844 persisted in the bacterial community until the end of the experiments on day 8, suggesting a low or negligible fitness cost to carrying the plasmid in this system, even in the absence of selection by antibiotic, as has been postulated previously (46).

Acquisition of multidrug-resistant plasmids with low fitness costs and high transfer rates by commensal bacteria in the chicken gut represents a manifest risk for the maintenance and dissemination of resistance in the food chain. Indeed, we observed plasmid transfer within 3 days, suggesting that a transient infection may be sufficient for plasmid dissemination to commensal bacteria. Furthermore, commensals have the potential to persist on farms, as they can reside within host animals, and in the environment, following excretion in feces. Additionally, wildlife such as rodents, which can be a significant reservoir of *Salmonella* on many poultry farms (47), may be colonized by multidrug-resistant commensals, further aiding the maintenance and dissemination of resistance in the environment. Once established in the commensal microbiota, the multidrug resistance plasmid can remain available for subsequent acquisition by pathogens following infection. This presents a potential risk for animal and public health, as treatment options may be limited if resistant zoonotic pathogens enter the food chain. Therefore, the *in vitro* gut model we describe, although not an exact facsimile of the gut, nevertheless provides a powerful and complex screening tool to assess and refine interventions that may serve to mitigate the spread of antibiotic resistance in the gut environment, such as synthetic fatty acids (66) or phage proteins (67). Furthermore, this system enables such controlled experimental *in vitro* studies to be undertaken before employing costly *in vivo* studies using higher animals and can help reduce the number of animals used for experiments.

In conclusion, the *in vitro* gut model we describe provides a valuable approximation of chicken cecal microbial diversity that enables investigation of the impact of colonization by a multidrug-resistant zoonotic pathogen and antibiotic administration. In particular, the model provides insight and demonstration into the dynamics of plasmid transfer and dissemination of antibiotic resistance to multiple commensal gut *E. coli* strains, which can be used to inform risk models studying dissemination of antibiotic resistance in bacteria from animals.

## MATERIALS AND METHODS

### Single-stage chemostat fermentation.

A parallel chemostat system comprising up to six single-stage fermentation vessels (20-ml culture volume) was used for the maintenance of the chicken cecal microbiota. The pH was maintained with an automatic pH controller (Fermac 260; Electrolab Biotech Limited, UK) at pH 5.8 to 6.0 to simulate the cecal pH, and the temperature was maintained at 41°C using a circulating water bath to maintain chicken body temperature. Anaerobic conditions, present in the gut, were maintained by continuous sparging with anaerobic gas mixture (80% N_2_, 10% CO_2_, and 10% H_2_) and continuously mixed with a magnetic flea. The chemostats were employed as single-stage closed fermentation vessels for the first 24 h after which continuous flow was started by adding fresh Viande-Leuvre culture medium (21) (flushed with anaerobic gas mixture) into the system with a peristaltic pump (VSPP; Electrolab Biotech Limited, UK) at a rate of 1.25 ml/h. Experiments were run for 8 days.

Cecal contents were recovered from the carcasses of 6-week-old White Leghorn chickens hatched from the eggs of specific-pathogen-free birds (Lohmann, Germany) and housed under experimental conditions in biosecure facilities. Chickens were reared on standard, nonmedicated chick feed (Lillico Attlee, UK) and provided with water *ad libitum*. The birds were obtained as carcasses on the day of slaughter. No regulated procedures were undertaken as part of this study, and therefore, no ethical approval was required.

For each experiment, cecal contents from five birds were pooled and mixed in an anaerobic cabinet with prereduced 0.1 M phosphate-buffered saline (PBS) (pH 7.2) at a ratio of 40:60 (vol/wt) (PBS-cecal contents) to create a slurry. For each vessel, 2 ml of this slurry was seeded into 18 ml of Viande-Leuvre culture medium. Cecal slurry was also used for quantitative bacteriology and stored at −20°C for subsequent DNA extraction. Samples (2 ml) were collected at 1- or 2-day intervals from each culture vessel and used for quantitative bacteriology, while 1 ml was centrifuged, with the subsequent cell pellet stored at −20°C in glycerol-PBS (50:50 [vol/vol]).

The monophasic S. Typhimurium isolate B3844 harboring plasmid pIFM3844, which carries the AMR genes *bla*_CTX-M1_, *sul2*, and *floR* (15), was employed to simulate colonization by a multidrug-resistant S. Typhimurium. To prepare cultures for inoculation into the gut model, strain B3844 was grown overnight in Luria-Bertani broth at 37°C with shaking at 300 rpm.

Four experiments were performed. Experiment 1 employed a single vessel seeded with cecal contents. Experiment 2 employed two vessels; cefotaxime (CTX) (Sigma, Gillingham, UK) was administered to one vessel on days 2 and 3, after the daily sample collection, at a final concentration of 20 mg/liter, in accordance with previous gut model experiments (20). Experiments 3 and 4 employed six vessels, which were divided into pairs as follows: two vessels received no *Salmonella*, two were inoculated with ~10^7^ CFU *Salmonella* at hour 0 (i.e., immediately after addition of the cecal slurry), and two were inoculated with ~10^7^ CFU *Salmonella* at 21 h after inoculation. The inoculating dose administered was determined by plating serial dilutions of the inoculum on LB agar. Cefotaxime was administered to three vessels in each experiment, one vessel of each pair, on days 2 and 3, to a final concentration of 20 mg/liter.

### Microbial profiling.

DNA was extracted from the cell pellets using the Gentra Puregene Yeast/Bact. kit (Qiagen, Crawley, UK). The cecal slurry was centrifuged and then washed three times with 0.1 M PBS (pH 7.2) before extraction. For each DNA preparation, the V4 and V5 regions of the 16S rRNA gene were amplified using bar-coded primers and sequenced by 454 pyrosequencing using the Roche 454 GS-FLX system, as described previously (48). The 16S rRNA sequence data were analyzed in the QIIME pipeline version 1.9.1 (25) installed on BioLinux 8 (49). AmpliconNoise was used for demultiplexing, denoising, and removing chimeras (50). Sequences were clustered into operational taxonomic units (OTUs) using UCLUST (51) with a 97% sequence identity threshold. Reads were aligned to the Greengenes core reference alignment (52) using PyNAST (53), and taxonomy was assigned with the Ribosomal Database Project classifier (minimum confidence of 80%) (54). FastTree was used for phylogenetic tree construction (55). Based on the number of sequences obtained per sample, the relative OTU abundance for each sample was determined at an even depth of 999 sequences per sample (randomly picked without replacement; singleton OTUs were excluded from this analysis), which was sufficient to describe the bacterial community (21).

The microbial profiles from all gut model experiments and the corresponding cecal samples were analyzed together, and the data were structured to allow comparison by time point, cefotaxime administration, *Salmonella* inoculation, and experiment number. The Chao1 and Shannon alpha diversity indices were calculated using QIIME, and significant differences (*P* ≤ 0.05) for the categorical variables were calculated in GraphPad Prism using the Mann-Whitney (two-tailed) test (cefotaxime administration) and the Kruskal-Wallis test (time point, *Salmonella* inoculation, and experiment number). Beta diversity was calculated using UniFrac (56), and the nonparametric statistical method Adonis (25) was employed with 999 permutations to identify significant differences (*P* ≤ 0.05) for the four variables using the weighted UniFrac distance matrix and both *P* and *R*^2^ values were reported. Three-dimensional principal-coordinate analysis plots of the weighted UniFrac distance matrix were visualized using EMPeror (57). To identify OTUs differing in abundance by these variables, the Mann-Whitney test (cefotaxime administration) or Kruskal-Wallis test (time point, *Salmonella* inoculation and experiment number) were used in QIIME (25), and OTUs with a *P* value of ≤0.05 (after Bonferroni’s correction for multiple tests) were classified as being significantly different.

### Quantitative bacteriology.

Quantitative bacteriology was performed for experiments 2, 3, and 4 using the method of Miles et al. (58), in which a 10-fold dilution series of the cecal inoculum and aliquots from gut model cultures were prepared in 0.1 M PBS (pH 7.2). Dilutions were plated onto Brilliance UTI (urinary tract infection) agar (Oxoid Limited, Basingstoke, UK) with or without cefotaxime (1 µg/ml, recommended by EUCAST [59] as the appropriate screening concentration for isolates that are ESBL producers) for enumeration of total presumptive *E. coli* and enterococci and to screen for presumptive ESBL-producing *E. coli*, identified by their chromogenic properties on plates containing cefotaxime. Presumptive *Salmonella* and *E. coli*, identified by their chromogenic properties on Rambach agar (Oxoid Limited, Basingstoke, UK) supplemented with 1 µg/ml cefotaxime, were also enumerated. Additionally, the cecal slurry used to seed the vessels was examined for *Salmonella* on Rambach agar without cefotaxime. Bacterial counts were analyzed using GraphPad Prism (version 6.04; GraphPad Software, Inc.).

Plasmid transfer rates via bacterial conjugation were calculated using the endpoint method of L. Simonsen et al. (26). The counts of total *Salmonella* (donors), total *E. coli* (recipients), and cefotaxime-resistant *E. coli* on Rambach plates (transconjugants) were used. An estimated exponential-phase growth rate of 2.2 was used for these calculations, based on the data presented by L. Simonsen et al. (26) on the growth rate of *E. coli* at 40°C.

### Isolate recovery, PCR, and whole-genome sequencing.

Representative isolates presumptively identified as *E. coli*, *Salmonella*, or *Enterococcus* were subcultured to purity and then identified to the species level by MALDI-TOF MS (60). DNA extracts were prepared from these isolates using Prepman Ultra (Life Technologies, Inc.) from overnight culture at 37°C on blood agar. The presence of *bla*_CTX-M1_ and plasmid pIFM3844 was determined by PCR using published primers (15, 61). DNA was extracted from selected isolates using the DNeasy kit (Qiagen, Crawley, UK) and Nextera XT libraries prepared for whole-genome sequencing (WGS) (Illumina, Lesser Chesterford, UK) sequenced on an Illumina MiSeq platform v2 using 2 × 250 bp paired-end protocol.

### Analysis of whole-genome sequences.

For each sequenced isolate, the raw sequences were filtered and trimmed using Trimmomatic (62), with the parameters for the minimum quality threshold equal to 20, a sliding window equal to 10, and a minimum sequence length equal to 36. The raw trimmed and filtered data were mapped onto the genome of the reference *E. coli* K-12 (GenBank accession number U00096) using SMALT (Sanger Institute). The published genomes of 12 *E. coli* isolates from poultry comprising commensal, ESBL-harboring, or avian pathogenic *E*. *coli* (APEC) isolates (see Table S7 in the supplemental material) were also mapped to *E*. *coli* K-12. Single nucleotide polymorphisms (SNPs) with respect to *E*. *coli* K-12 were calculated using SAMTOOLS software (63, 64). SNPs were filtered using the quality thresholds of minimum coverage equal to 4, minimum proportion of raw sequences agreeing with the SNP call equal to 80%, and SAMTOOLS SNP quality score of >150. A maximum likelihood phylogenetic tree using the SNPs located within regions present for all the strains was constructed using RAxML (65).

10.1128/mBio.00777-17.9 TABLE S7  Published whole-genome sequences of poultry *E. coli* used in the phylogenetic tree. Download TABLE S7, XLSX file, 0.01 MB. Copyright © 2017 Card et al. 2017 Card et al. This content is distributed under the terms of the Creative Commons Attribution 4.0 International license.

The antimicrobial resistance (AMR) gene content of the isolates was assessed using SeqFinder, as described previously (27). Isolate sequence type (ST) was determined by extracting the seven housekeeping genes of the Achtman multilocus sequence type (MLST) scheme (*adk*, *fumC*, *gyrB*, *icd*, *mdh*, *purA*, and *recA*) and interrogation of the PubMLST database (http://pubmlst.org/mlst/).

### Accession number(s).

The 16S rRNA sequences and whole-genome sequences were deposited in the European Nucleotide Archive under study accession number PRJEB18652.
