# Supplementary material for: An In Vitro Chicken Gut Model Demonstrates Transfer of a Multidrug Resistance Plasmid from Salmonella to Commensal Escherichia coli
Source: mBio. 2017 Jul 18;8(4):e00777-17. doi: 10.1128/mBio.00777-17 (PMC5516254; doi:10.1128/mBio.00777-17)

Figure S1. a) Chao1 alpha diversity plots for experiment number, time-point, *Salmonella* inoculation, and cefotaxime administration. Significance of differences in Chao1 indices by each categorical variable are given.

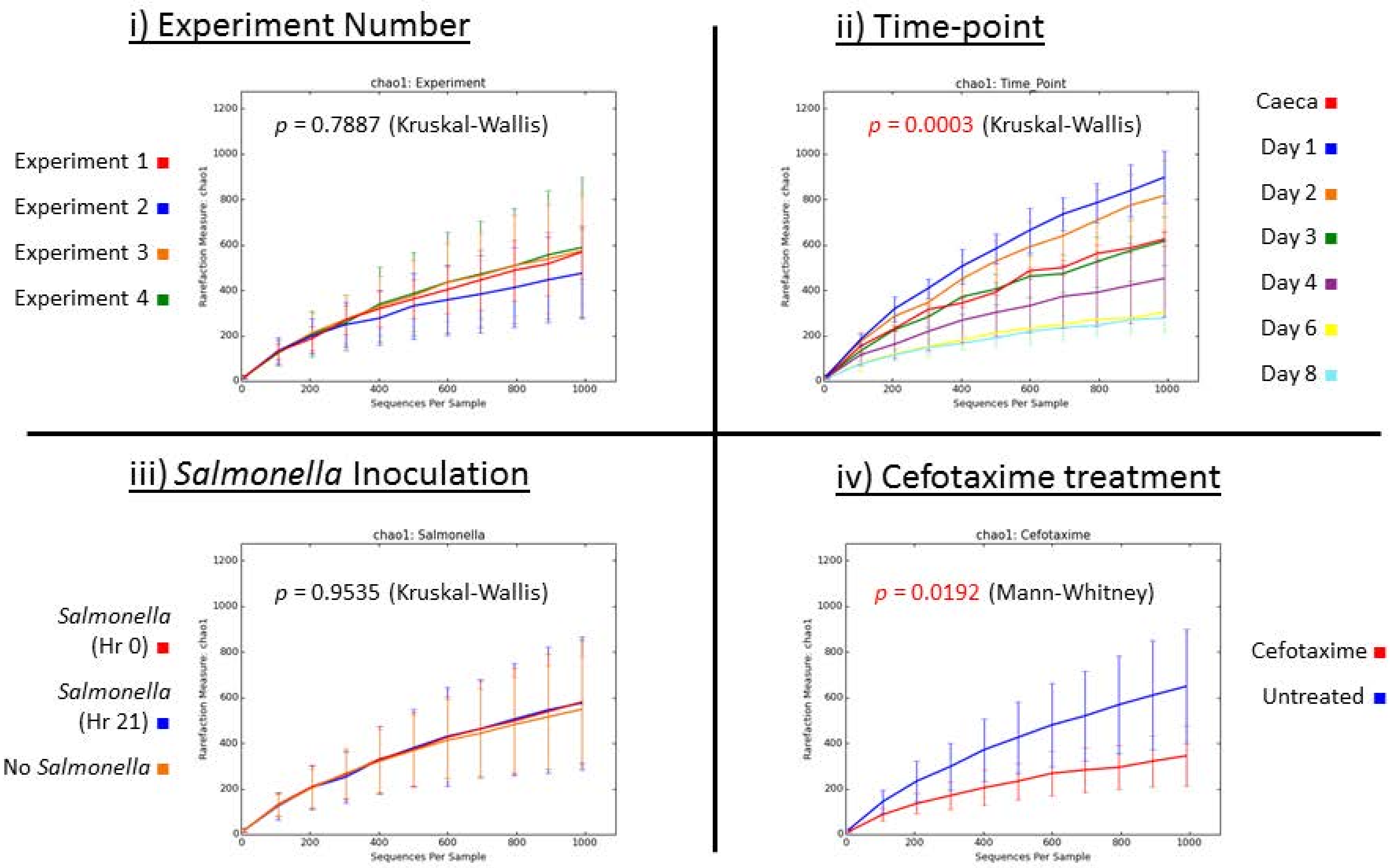

Figure S1 b) Shannon alpha diversity plots for experiment number, time-point, *Salmonella* inoculation, and cefotaxime administration. Significance of differences in Shannon indices by each categorical variable are given.

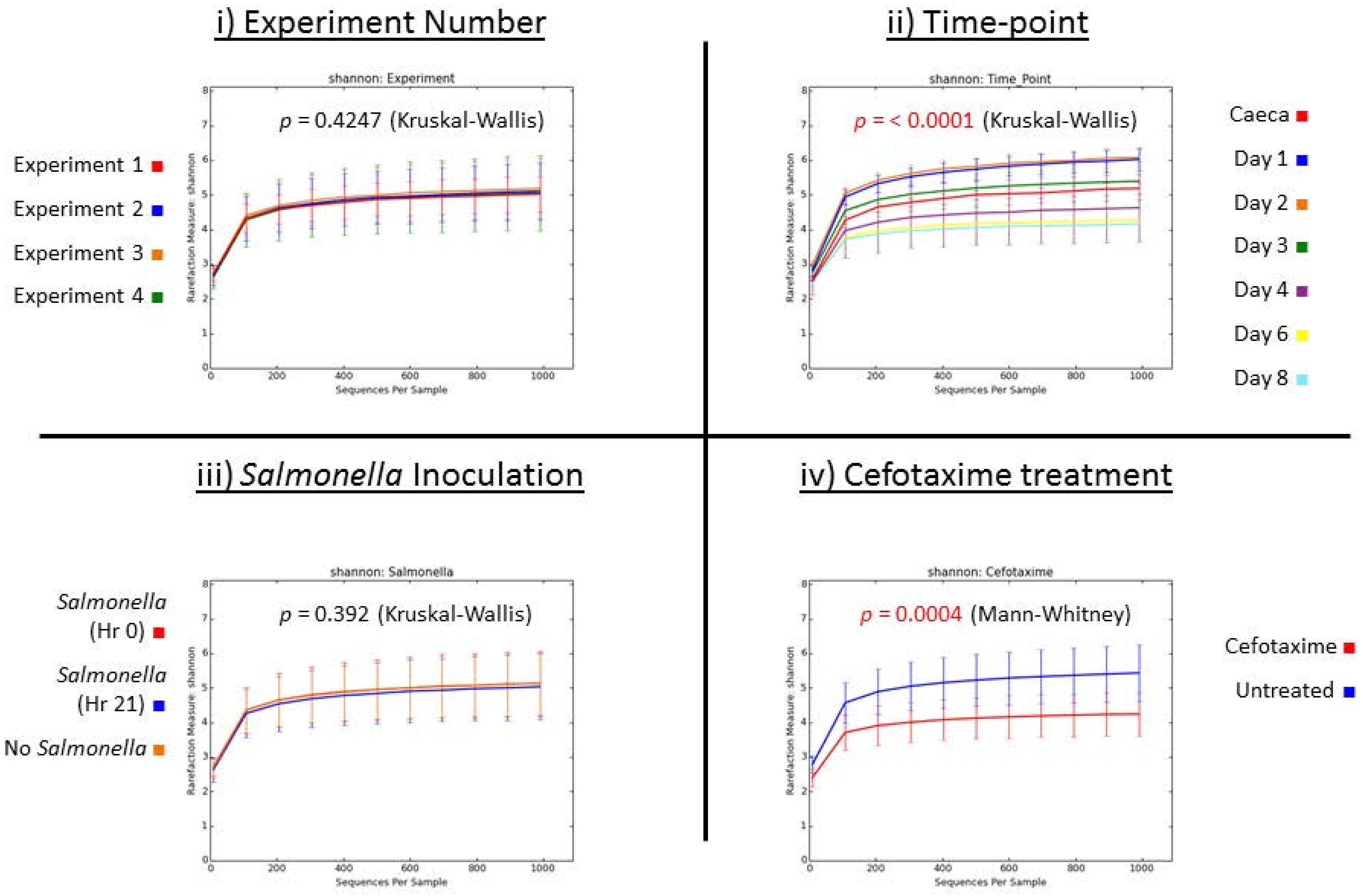

Supplement: FIG S1 [file mbo003173381sf1.pdf]
