# Supplementary figures and images for: An In Vitro Chicken Gut Model Demonstrates Transfer of a Multidrug Resistance Plasmid from Salmonella to Commensal Escherichia coli
Source: mBio. 2017 Jul 18;8(4):e00777-17. doi: 10.1128/mBio.00777-17 (PMC5516254; doi:10.1128/mBio.00777-17)

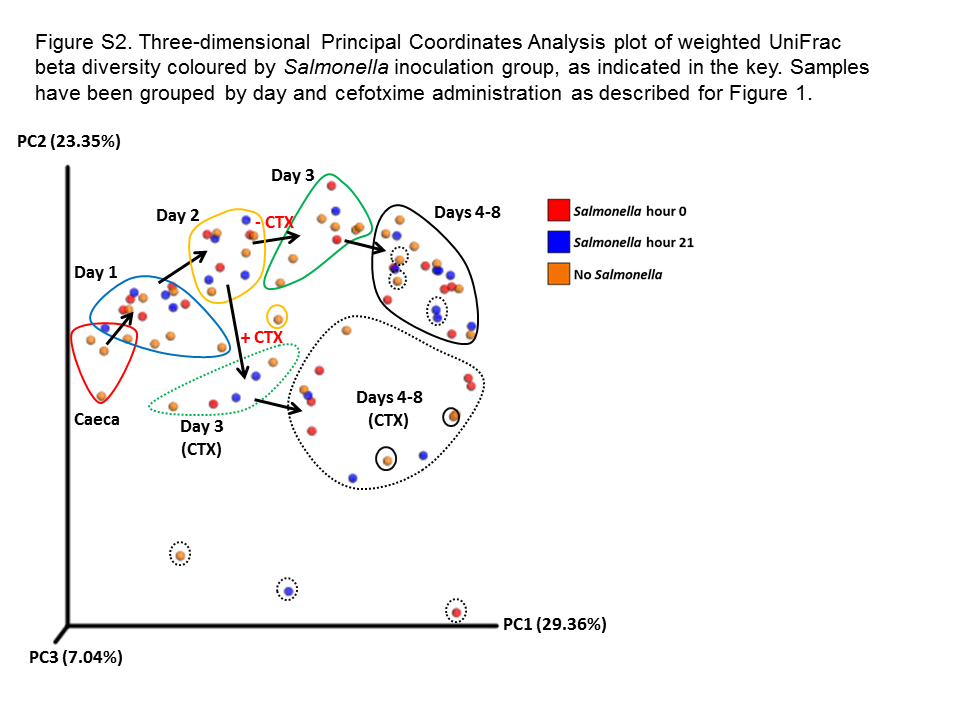

Supplement: FIG S2 [file mbo003173381sf2.tif]
